# 1-Benz­yloxy-4-chloro­benzene

**DOI:** 10.1107/S160053680905096X

**Published:** 2009-12-04

**Authors:** Guo-Xi Wang

**Affiliations:** a Department of Chemical and Environmental Engineering, Anyang Institute of Technology, Anyang 455000, People’s Republic of China

## Abstract

In the title compound, C_13_H_11_ClO, the two benzene rings are close to coplanar, making a dihedral angle of 3.4 (1)° The crystal structure is stabilized by weak C—H⋯π inter­actions involving both benzene rings.

## Related literature

For the chemistry and crystal structures of halogenated aromatic ether derivatives, see: Liu *et al.* (2006 ▶); Shen *et al.* (2003 ▶). 
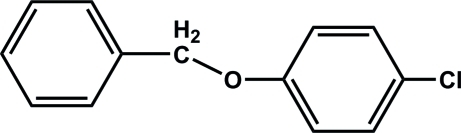


## Experimental

### 

#### Crystal data

C_13_H_11_ClO
*M* *_r_* = 218.67
Orthorhombic, 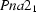

*a* = 11.485 (2) Å
*b* = 13.033 (3) Å
*c* = 7.3333 (15) Å
*V* = 1097.7 (4) Å^3^
*Z* = 4
Mo *K*α radiation
μ = 0.32 mm^−1^
*T* = 298 K
0.4 × 0.35 × 0.2 mm

#### Data collection

Rigaku Mercury2 diffractometer
Absorption correction: multi-scan (*CrystalClear*; Rigaku, 2005 ▶) *T*_min_ = 0.881, *T*_max_ = 0.940
10943 measured reflections
2523 independent reflections
2189 reflections with *I* > 2σ(*I*)
*R*_int_ = 0.039

#### Refinement

*R*[*F* ^2^ > 2σ(*F* ^2^)] = 0.051
*wR*(*F* ^2^) = 0.115
*S* = 1.16
2523 reflections
137 parameters
1 restraint
H-atom parameters constrained
Δρ_max_ = 0.31 e Å^−3^
Δρ_min_ = −0.48 e Å^−3^
Absolute structure: Flack (1983 ▶), 1161 Friedel pairs
Flack parameter: −0.08 (9)

### 

Data collection: *CrystalClear* (Rigaku, 2005 ▶); cell refinement: *CrystalClear*; data reduction: *CrystalClear*; program(s) used to solve structure: *SHELXS97* (Sheldrick, 2008 ▶); program(s) used to refine structure: *SHELXL97* (Sheldrick, 2008 ▶); molecular graphics: *SHELXTL* (Sheldrick, 2008 ▶); software used to prepare material for publication: *SHELXTL*.

## Supplementary Material

Crystal structure: contains datablocks I, global. DOI: 10.1107/S160053680905096X/ci2976sup1.cif

Structure factors: contains datablocks I. DOI: 10.1107/S160053680905096X/ci2976Isup2.hkl

Additional supplementary materials:  crystallographic information; 3D view; checkCIF report

## Figures and Tables

**Table 1:** Hydrogen-bond geometry (Å, °)

*D*—H⋯*A*	*D*—H	H⋯*A*	*D*⋯*A*	*D*—H⋯*A*
C1—H1⋯*Cg*2^i^	0.93	2.81	3.570 (3)	140
C10—H10⋯*Cg*1^i^	0.93	2.88	3.624 (3)	138

Symmetry code: (i) 
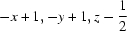
. *Cg*1 and *Cg*2 are the centroids of the C1–C6 and C8–C13 rings, respectively.

**Fig. 1.**
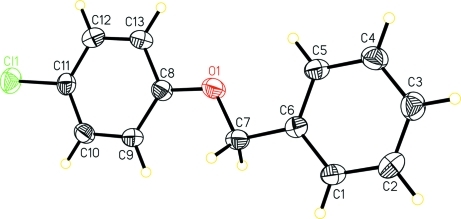
A view of the title compound with the atomic numbering scheme. Displacement ellipsoids are drawn at the 30% probability level.

## supplementary crystallographic information

### Comment

Halogenated aromatic ether derivatives have found wide range of applications in industry and coordination chemistry as ligands. They are also used in medicine as drugs, such as antibiotics. Recently, a series of halogenated aromatic ether compounds have been reported (Liu *et al.*, 2006; Shen *et al.*, 2003). As an extension of these work on the structural characterization, we report here the crystal structure of the title compound, 1-(benzyloxy)-4-chlorobenzene.

The crystal data show that in the title compound (Fig.1), the two benzene rings are essentially coplanar and twisted from each other by a dihedral angle of 3.4 (1)°. All bond lengths are within the normal range. The crystal structure is stabilized by weak C—H···π interactions.

### Experimental

The commercial 1-(benzyloxy)-4-chlorobenzene (3 mmol, 648 mg) was dissolved in chloroform (20 ml). The solvent was slowly evaporated in air affording colourless block-shaped crystals of the title compound suitable for X-ray analysis.

### Refinement

All H atoms were fixed geometrically and treated as riding with C–H = 0.93 Å (aromatic) or 0.97 Å (methylene) and *U*_iso_(H) =1.2U_eq_(C).

### Crystal data

**Table:**

C_13_H_11_ClO	*F*(000) = 456
*M**_r_* = 218.67	*D*_x_ = 1.323 Mg m^−^^3^
Orthorhombic, *P**n**a*2_1_	Mo *K*α radiation, λ = 0.71073 Å
Hall symbol: P 2c -2n	Cell parameters from 2189 reflections
*a* = 11.485 (2) Å	θ = 3.1–27.5°
*b* = 13.033 (3) Å	µ = 0.32 mm^−^^1^
*c* = 7.3333 (15) Å	*T* = 298 K
*V* = 1097.7 (4) Å^3^	Bock, colourless
*Z* = 4	0.4 × 0.35 × 0.2 mm

### Data collection

**Table:**

Rigaku Mercury2 diffractometer	2523 independent reflections
Radiation source: fine-focus sealed tube	2189 reflections with *I* > 2σ(*I*)
graphite	*R*_int_ = 0.039
Detector resolution: 13.6612 pixels mm^-1^	θ_max_ = 27.5°, θ_min_ = 3.1°
ω scans	*h* = −14→14
Absorption correction: multi-scan (*CrystalClear*; Rigaku, 2005)	*k* = −16→16
*T*_min_ = 0.881, *T*_max_ = 0.940	*l* = −9→9
10943 measured reflections	

### Refinement

**Table:**

Refinement on *F*^2^	Hydrogen site location: inferred from neighbouring sites
Least-squares matrix: full	H-atom parameters constrained
*R*[*F*^2^ > 2σ(*F*^2^)] = 0.051	*w* = 1/[σ^2^(*F*_o_^2^) + (0.0637*P*)^2^ + 0.0118*P*] where *P* = (*F*_o_^2^ + 2*F*_c_^2^)/3
*wR*(*F*^2^) = 0.115	(Δ/σ)_max_ = 0.001
*S* = 1.16	Δρ_max_ = 0.31 e Å^−^^3^
2523 reflections	Δρ_min_ = −0.48 e Å^−^^3^
137 parameters	Extinction correction: *SHELXL97* (Sheldrick, 2008), Fc^*^=kFc[1+0.001xFc^2^λ^3^/sin(2θ)]^-1/4^
1 restraint	Extinction coefficient: 0.073 (6)
Primary atom site location: structure-invariant direct methods	Absolute structure: Flack (1983), 1161 Friedel pairs
Secondary atom site location: difference Fourier map	Flack parameter: −0.08 (9)

### Special details

**Table:**

Geometry. All e.s.d.'s (except the e.s.d. in the dihedral angle between two l.s. planes) are estimated using the full covariance matrix. The cell e.s.d.'s are taken into account individually in the estimation of e.s.d.'s in distances, angles and torsion angles; correlations between e.s.d.'s in cell parameters are only used when they are defined by crystal symmetry. An approximate (isotropic) treatment of cell e.s.d.'s is used for estimating e.s.d.'s involving l.s. planes.
Refinement. Refinement of *F*^2^ against ALL reflections. The weighted *R*-factor *wR* and goodness of fit *S* are based on *F*^2^, conventional *R*-factors *R* are based on *F*, with *F* set to zero for negative *F*^2^. The threshold expression of *F*^2^ > σ(*F*^2^) is used only for calculating *R*-factors(gt) *etc*. and is not relevant to the choice of reflections for refinement. *R*-factors based on *F*^2^ are statistically about twice as large as those based on *F*, and *R*- factors based on ALL data will be even larger.

### Fractional atomic coordinates and isotropic or equivalent isotropic displacement parameters (Å^2^)

**Table:**

	*x*	*y*	*z*	*U*_iso_*/*U*_eq_	
Cl1	0.76032 (5)	0.10633 (4)	0.35041 (15)	0.0662 (2)	
C11	0.72273 (17)	0.23611 (14)	0.3557 (3)	0.0450 (4)	
C6	0.53613 (16)	0.69777 (14)	0.3347 (3)	0.0400 (4)	
C5	0.62546 (18)	0.74952 (15)	0.4233 (3)	0.0472 (5)	
H5	0.6896	0.7134	0.4663	0.057*	
C12	0.79431 (18)	0.30526 (17)	0.4443 (3)	0.0477 (5)	
H12	0.8636	0.2833	0.4969	0.057*	
O1	0.63588 (13)	0.54166 (11)	0.3941 (3)	0.0610 (5)	
C8	0.65937 (17)	0.43950 (15)	0.3727 (3)	0.0443 (4)	
C4	0.6193 (2)	0.85449 (18)	0.4478 (4)	0.0555 (6)	
H4	0.6791	0.8885	0.5085	0.067*	
C10	0.62149 (18)	0.26770 (16)	0.2733 (3)	0.0483 (5)	
H10	0.5747	0.2206	0.2125	0.058*	
C13	0.76238 (17)	0.40660 (17)	0.4542 (3)	0.0468 (5)	
H13	0.8096	0.4533	0.5153	0.056*	
C9	0.58956 (18)	0.37006 (16)	0.2814 (3)	0.0471 (5)	
H9	0.5213	0.3920	0.2254	0.056*	
C7	0.53952 (18)	0.58411 (16)	0.3009 (3)	0.0480 (5)	
H7A	0.4681	0.5527	0.3441	0.058*	
H7B	0.5464	0.5708	0.1712	0.058*	
C2	0.4370 (2)	0.85864 (19)	0.2956 (4)	0.0603 (6)	
H2	0.3736	0.8953	0.2514	0.072*	
C1	0.44145 (19)	0.75356 (17)	0.2727 (3)	0.0502 (5)	
H1	0.3803	0.7199	0.2151	0.060*	
C3	0.5254 (2)	0.90919 (16)	0.3831 (4)	0.0587 (6)	
H3	0.5221	0.9799	0.3987	0.070*	

### Atomic displacement parameters (Å^2^)

**Table:**

	*U*^11^	*U*^22^	*U*^33^	*U*^12^	*U*^13^	*U*^23^
Cl1	0.0683 (4)	0.0468 (3)	0.0835 (4)	0.0071 (2)	0.0070 (4)	−0.0037 (4)
C11	0.0476 (10)	0.0429 (9)	0.0443 (10)	0.0007 (8)	0.0080 (10)	−0.0011 (12)
C6	0.0392 (9)	0.0465 (10)	0.0344 (9)	−0.0003 (7)	0.0012 (8)	0.0031 (8)
C5	0.0399 (10)	0.0505 (12)	0.0514 (13)	−0.0016 (8)	−0.0072 (8)	0.0054 (10)
C12	0.0396 (10)	0.0555 (13)	0.0479 (11)	0.0008 (9)	−0.0008 (10)	0.0078 (10)
O1	0.0647 (9)	0.0431 (7)	0.0752 (11)	0.0053 (6)	−0.0318 (8)	−0.0081 (8)
C8	0.0474 (10)	0.0428 (9)	0.0426 (10)	−0.0012 (8)	−0.0041 (9)	−0.0006 (10)
C4	0.0502 (12)	0.0533 (12)	0.0631 (14)	−0.0104 (10)	−0.0051 (11)	−0.0005 (11)
C10	0.0460 (11)	0.0518 (11)	0.0471 (10)	−0.0068 (9)	−0.0001 (10)	−0.0095 (10)
C13	0.0452 (11)	0.0484 (12)	0.0469 (12)	−0.0071 (8)	−0.0093 (10)	0.0001 (9)
C9	0.0399 (10)	0.0547 (11)	0.0466 (11)	0.0026 (8)	−0.0082 (9)	−0.0061 (10)
C7	0.0440 (10)	0.0511 (11)	0.0488 (13)	−0.0004 (8)	−0.0100 (9)	−0.0008 (9)
C2	0.0518 (12)	0.0606 (13)	0.0686 (16)	0.0148 (11)	−0.0061 (11)	0.0069 (12)
C1	0.0395 (10)	0.0578 (13)	0.0535 (11)	0.0006 (8)	−0.0077 (10)	−0.0018 (10)
C3	0.0615 (14)	0.0442 (10)	0.0705 (17)	0.0003 (9)	0.0027 (13)	0.0021 (12)

### Geometric parameters (Å, °)

**Table:**

Cl1—C11	1.7460 (19)	C4—C3	1.377 (3)
C11—C10	1.374 (3)	C4—H4	0.93
C11—C12	1.382 (3)	C10—C9	1.385 (3)
C6—C1	1.385 (3)	C10—H10	0.93
C6—C5	1.389 (3)	C13—H13	0.93
C6—C7	1.502 (3)	C9—H9	0.93
C5—C4	1.382 (3)	C7—H7A	0.97
C5—H5	0.93	C7—H7B	0.97
C12—C13	1.373 (3)	C2—C3	1.370 (3)
C12—H12	0.93	C2—C1	1.381 (3)
O1—C8	1.367 (2)	C2—H2	0.93
O1—C7	1.413 (2)	C1—H1	0.93
C8—C9	1.382 (3)	C3—H3	0.93
C8—C13	1.393 (3)		
			
C10—C11—C12	120.99 (19)	C12—C13—C8	120.0 (2)
C10—C11—Cl1	119.34 (16)	C12—C13—H13	120.0
C12—C11—Cl1	119.67 (17)	C8—C13—H13	120.0
C1—C6—C5	118.59 (18)	C8—C9—C10	119.85 (19)
C1—C6—C7	118.95 (17)	C8—C9—H9	120.1
C5—C6—C7	122.45 (17)	C10—C9—H9	120.1
C4—C5—C6	120.25 (19)	O1—C7—C6	109.07 (15)
C4—C5—H5	119.9	O1—C7—H7A	109.9
C6—C5—H5	119.9	C6—C7—H7A	109.9
C13—C12—C11	119.55 (19)	O1—C7—H7B	109.9
C13—C12—H12	120.2	C6—C7—H7B	109.9
C11—C12—H12	120.2	H7A—C7—H7B	108.3
C8—O1—C7	118.71 (15)	C3—C2—C1	120.4 (2)
O1—C8—C9	125.35 (17)	C3—C2—H2	119.8
O1—C8—C13	114.72 (17)	C1—C2—H2	119.8
C9—C8—C13	119.93 (19)	C2—C1—C6	120.7 (2)
C3—C4—C5	120.5 (2)	C2—C1—H1	119.7
C3—C4—H4	119.7	C6—C1—H1	119.7
C5—C4—H4	119.7	C2—C3—C4	119.5 (2)
C11—C10—C9	119.62 (19)	C2—C3—H3	120.2
C11—C10—H10	120.2	C4—C3—H3	120.2
C9—C10—H10	120.2		
			
C1—C6—C5—C4	−0.2 (3)	O1—C8—C9—C10	−178.3 (2)
C7—C6—C5—C4	178.7 (2)	C13—C8—C9—C10	0.9 (3)
C10—C11—C12—C13	1.6 (3)	C11—C10—C9—C8	−0.3 (3)
Cl1—C11—C12—C13	−177.57 (17)	C8—O1—C7—C6	−176.12 (18)
C7—O1—C8—C9	−7.0 (3)	C1—C6—C7—O1	−173.0 (2)
C7—O1—C8—C13	173.7 (2)	C5—C6—C7—O1	8.0 (3)
C6—C5—C4—C3	−0.7 (4)	C3—C2—C1—C6	−1.2 (4)
C12—C11—C10—C9	−1.0 (3)	C5—C6—C1—C2	1.2 (3)
Cl1—C11—C10—C9	178.22 (17)	C7—C6—C1—C2	−177.8 (2)
C11—C12—C13—C8	−1.0 (3)	C1—C2—C3—C4	0.2 (4)
O1—C8—C13—C12	179.0 (2)	C5—C4—C3—C2	0.7 (4)
C9—C8—C13—C12	−0.3 (3)		

### Hydrogen-bond geometry (Å, °)

**Table:**

*D*—H···*A*	*D*—H	H···*A*	*D*···*A*	*D*—H···*A*
C1—H1···Cg2^i^	0.93	2.81	3.570 (3)	140
C10—H10···Cg1^i^	0.93	2.88	3.624 (3)	138

Symmetry codes: (i) −*x*+1, −*y*+1, *z*−1/2.
